# Effects of Cyclic Tensile Strain on Oxidative Stress and the Function of Schwann Cells

**DOI:** 10.1155/2018/5746525

**Published:** 2018-06-10

**Authors:** Shuang Li, Xiaolei Sun, Xinlong Ma

**Affiliations:** Tianjin Hospital, Jiefang Nan Road 406, Tianjin 300211, China

## Abstract

Schwann cells (SCs) are significant due to the way in which they sustain and myelinate axons within the peripheral nervous system (PNS). This study has investigated the effect of cyclic tensile strain (CTS) on the oxidative stress and function of SCs derived from the sciatic nerves of an infant rat population. A group of 20 6-day-old Wistar rats was selected, and SCs were separated from the sciatic nerve. The SCs then underwent a 6-hour period of cyclical straining, and ElectroForce 3200 in combination with the BioDynamic chamber was employed to apply 0% and 5% strains at a 0.25 Hz frequency. The results showed that the control group suffered higher oxidative stress than that in 5% strains group (*P*<0.05). The results RT-PCR analysis indicated a correlation between 5% CTS and a reduction in Netrin-1 expression (*P*<0.05). Furthermore, there was a significant upregulation in NGF, GDNF, and Slit-2 gene expression (*P*<0.05). Finally, the results showed that CTS stimulate SCs by increasing the expression of nerve-oriented factors, and these importantly caused the decrease of oxidative stress, reconstruction of cell skeleton, the promotion of axonal regrowth, and the augmentation of nerves.

## 1. Introduction

The key point of dissimilarity when comparing the central nervous system (CNS) to the peripheral nervous system (PNS) relates to the latter's inherent ability to spontaneously regenerate. Research demonstrates that despite how the function of peripheral nerves is impaired upon sustaining an injury, they display a capacity for regrowth and, moreover, for reconnection to their targets [1, 2]. Schwann cells (SCs) are important due to the way in which they sustain and myelinate axons within the PNS, and immature SCs—produced via intermediate SC precursors from the neural crest—are distinct from their mature counterparts in view of the capacity they have for cellular differentiation [3, 4]. Immature SCs can morph into cells that include both myelin-forming and non-myelin-forming variations and are critical to the successful function and regrowth of peripheral nerves. Because SCs are structurally and functionally central to the PNS and are a key contributor to the process of neuroregeneration for peripheral nerves, the formulation of numerous strategic approaches to the enhancement of peripheral nerve regeneration currently revolves around their investigation [5, 6]. Therefore, much recent academic research has focused on determining the effective pharmaceutical agents that positively influence SC behaviour and functions.

With regard to wound healing processes, mechanical stimuli are linked to numerous cytological and molecular biological phenomena. Several studies have illuminated the positive role that tensile strength and frequency, when operating within a suitable range, play in the healing of neurological function, and evidence shows that a minor portion of the axon induces axon extension [7–9]. The imbalance of oxygen radical systems involves the excessive production of reactive oxygen species (ROS), including super oxygen anion (O_2_^−^), hydrogen peroxide (H_2_O_2_), hydroxyl free radicals (-OH), and malondialdehyde (MDA), accompanied by a recession in the free radical-scavenging function of superoxide dismutase (SOD), catalase (CAT), and glutathion peroxidase (GPx), and increasing the enhancement of lipid peroxidation, which is a vital factor in tissue damage observed in many diseases. It was found that mitochondrial dysfunction and corresponding oxidative stress generation were induced in the chemotherapy, which mediate peripheral nerve damage [10].

A decision was made to conduct research on this specific topic, as a literature review of existing academic works indicated a lack of in-depth knowledge of the impact of cyclic tensile strain (CTS) on SCs. Hence, this study will evaluate the ways in which CTS affects the oxidative stress, behaviour, and functions of primary SC, and the potential contributory role played by related genes. This research constitutes the initial systemic examination to address the extent to which CTS is biologically significant with respect to these variables.

## 2. Materials and Methods

### 2.1. Animals

Prior to being conducted, every course of experimentation outlined in this study received approval from an Ethical Committee at Tianjin Hospital (Tianjin, China). Thus, each phase of the research reported in this paper conforms to the relevant stipulations and provisions mandated by the committee. The animal specimens drawn on for experimental purposes, 20 6-day-old Wistar rats bought from Tianjin Laboratory Animal Public Service Center, could eat and drink freely, and they were held in a measured laboratory environment. This environment maintained a light/dark cycle of 12 hours/12 hours (with light activation at 06:00), a temperature of 22 ± 2.5°C, and an air humidity of 55-65%.

### 2.2. Isolation and Culture of SCs

In accordance with the processes overviewed in previous reports [11, 12], the acquisition of SCs was conducted by targeting the sciatic nerves of the 6-day-old Wistar rats. Briefly, the bilateral sciatic nerves and brachial plexuses of twenty 6-day-old Wistar rats were harvested and then washed with PBS (pH = 7.4). After separating the epineurium, 0.03% type I collagenase and 0.25% trypsin were used to cut the nerve into less than 1 mm discrete clusters in 10 ml PBS for 30 min at 37°C. In turn, the growth of the SCs was carried out in Dulbecco's modified Eagle's medium/F-12 Ham nutrient mixture (DMEM/F-12; Gibco, USA), which contained the following: 10% fetal bovine serum (FBS; Gibco); 1% penicillin-streptomycin solution (Gibco); and 2 mm (Sigma-Aldrich). Prior to the below-mentioned experimentation commencing, the researcher drew on the first passage (P1) cells, which were identified through S-100 and anti-glial fibrillary acidic protein (GFAP) immunostaining. Subsequently, seeding of the SCs took place in the plates, and these were sustained using a serum medium for a period of 24 hours. Finally, the culture medium was substituted with a serum medium incorporating one of the following: (1) 40% (1,400,000 plate- lets/mL); (2) 20% (700,000 platelets/mL); (3) 10% (350,000 platelets/mL); (4) 5% (175,000 platelets/mL); or (5) 2.5% (87,500 platelets/mL) PRP (v/v). It is important to note that control cultures were conducted in the same method without the presence of PRP.

### 2.3. Cyclic Tensile Strain

ElectroForce 3200 and the BioDynamic chamber (BOSS) were employed to function as the loading instrument, and following the growth of the cells under the previously discussed controlled conditions, the products were divided into two sets. The first set was subjected to 5% equibiaxial CTS with a 0.25 Hz (involving one second elongation and one second relaxation). This incorporated the usage of loading posts with a diameter of 25 mm, for the duration of 24 hours. The second set of cells was not subject to CTS during this time.

### 2.4. Immunocytochemical Staining Identification of SCs

The* in vitro *amplification of the P2 generation of SCs was carried out, and incubation was conducted on 0.01% polylysine on the coverslip. At the point where the SCs accounted for 70-80% of the plate, 4% paraformaldehyde was applied to the coverslip for a period of 15 minutes, and this was subsequently subjected to 3 3-minute periods of washing with 0.02 mol/L PBS. Following this, the SCs were washed with 3% H_2_O_2_ diluted 50 times in methanol, which resulted in the inactivation of the peroxidase for a period of 15 minutes. After the cells had been through the three-time washing process, they were incubated for 5 minutes at room temperature with a 10% conventional sheep serum blocking antibody. This process included the mouse anti-rat S-100 primary anti-mouse antibody (Titer 1:50). The SCs were controlled overnight at a temperature of 4°C, after which a goat anti-mouse secondary antibody labelled with biotin was added. Following incubation for a period of 2 hours at room temperature, the SCs were further incubated with DAB for a period of 15 minutes. Once haematoxylin staining had been carried out, a 100-fold optical microscope was utilised to assess the morphological features of the SCs, and it was observed that the tan was positive. Finally, in order to evaluate the histological elements of P3 SCs, a 100-fold inverted phase-contrast microscope was used.

### 2.5. Cell Migration Studies

In accordance with the methods of Kashani et al. [13], 6.5 mm Transwell chambers with 8 mm pores were employed to examine the ability of the PRP samples to stimulate the migration of rat SCs. The chambers at the bottom of the device were filled with a serum-free medium, and the higher surface of every membrane was sterilised with cotton balls following a 12-hour period of incubation at 37°C and a humidified 5% CO atmosphere. Consequently, the migrated cells attached to the membrane's lower side, and 4% paraformaldehyde was used to facilitate fixing. Finally, straining and counting processes were conducted.

### 2.6. Real-Time PCR

The TRIzol reagent (Takara Bio, Japan) was used to isolate the total of RNA and then treated with DNase I (Invitrogen, USA) according to the manufacture's instruction. The total number of RNA in the sample was measured in the 260 nm spectrum. Subsequently, the assessment of the expression of the NGF, GDNF, Netrin-1, and Slit-2 genes was achieved by employing the SYBR Premix Ex Taq (Takara Bio). The normalisation of the amount of mRNA in the cultures was facilitated by using the reference household gene, GAPDH, and the sequences for the primers presented as follows were evaluated closely in terms of their specificity: NGF, forward 5′-TCAACAGGACTCACAGGAGCA-3′, reverse 5′-GGTCTTATC TCCAACCCACACAC-3′ [3]; GDNF, forward 5′-CAGAGGGAAA GGTCGCAGAG-3′, reverse 5′-ATCAGTTCCTCCTTGGTTTCGTAG-3′ [3]; Netrin-1, forward 5′-CTTCTGCGGCAGGCGGACAGAT-3′, reverse 5′-ACGCGTTGCAGAGGTGGCACGA-3′ [14]; Slit-2, forward 5′-AACTTGTACTGCGACTGCCA-3′, reverse 5′-TCCTCATCACTGCAGACAAACT-3′ [15]; GAPDH, forward 5′-GGCACAGTCAAGGCTGAG AATG-3′, reverse 5′-ATGGTGGTGAAGACGCCAGTA-3′ [3]. Finally, relative expression refers to the ratio of target gene to control gene by means of Ct = (Cttarget − Ct*β*-actin) treatment − (Cttarget −Ct*β*-actin) control [16].

### 2.7. Total Antioxidant Activities

Based on the existing literature, ABTS*∗*+ radical-scavenging activity was identified. It should be noted that ABTS*∗*+ were pregenerated by combining 5 mL of a 4.9-mM potassium persulfate solution to 5 mL of a 14-mM ABTS solution, which was then maintained for a 16-hour period under minimal light conditions. Following this, the cells were combined with the pregenerated ABTS solution, which was appropriately diluted with distilled water, thereby giving rise to an absorbance of 0.70 at 734 nm. This was subsequently employed to serve as the antioxidant assay, while the reference compound was ascorbic acid (50 *μ*g/mL). In turn, the 950* μ*L of the ABTS solution was combined with this, and this underwent vertexing for a 10-second period. Within 6 minutes, the fall in absorbance was identified at 734 nm, relying on distilled water as a blank, on an ELICO (SL-150) UV-visible spectrophotometer. The enzymatic defence system was constituted of superoxide dismutase (SOD), catalase (CAT), and glutathione peroxidase (GPx), each of which underwent analysis based on the user manual (with commercial kits produced from the Nanjing Jiancheng Biotechnology Company, Nanjing, China).

### 2.8. Statistical Analysis

The data collected from the experiments was logged as means ± SEM, and the analysis focused on the use of Student's* t*-test with SPSS v 22.0. Differences were regarded as being statistically significant at* P<*0.05.

## 3. Results

**Figure 1:**
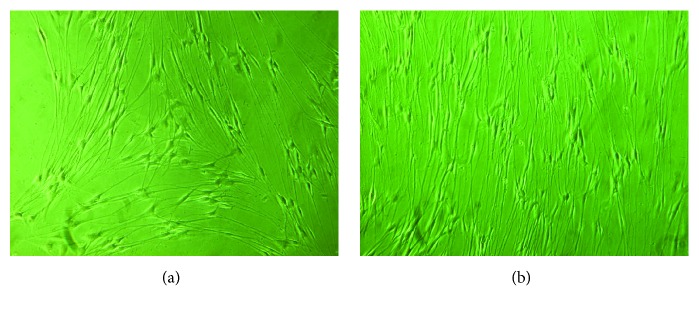
(a) Control group and (b) 5% CTS group, phase-contrast microscopic appearance of cultured SCs. In the control group, there is ambiguous polarity of the SCs, whereas conversely, in the 5% CTS group, the quantity and spread are rising and the SCs are displaying strong polarity.

Following immunocytochemical staining of SCs, the positive rate of SCs was 96.3 ± 2.7% under 100-fold optical microscope. Next, the morphology of the SCs was assessed under a microscope for both the control group and the 5% CTS group. SC polarity was not immediately apparent for the control group (Figure 1); however, following the 5% CTS, the number and proliferation of SCs increases and the SCs display strong polarity.

**Figure 2:**
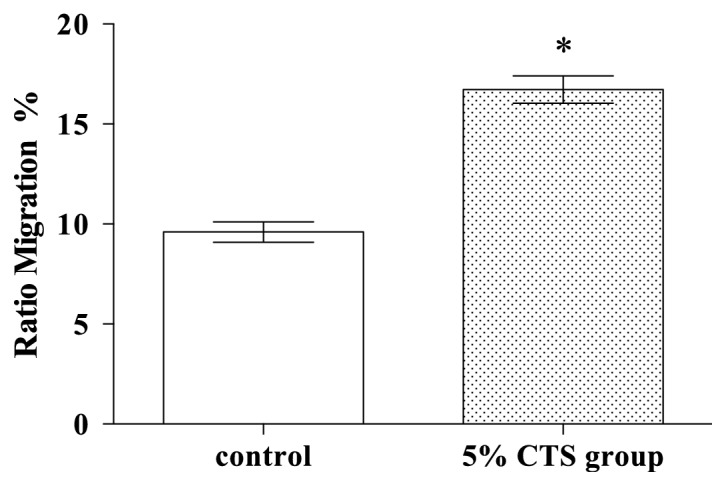
The migration of SCs for the control and 5% CTS groups. *∗* indicates that 5% CTS group differs significantly from the control group (*P*<0.05).

As the motility and migration of SCs are a critical function in the neuroregeneration of peripheral nerves, it was deemed appropriate to investigate the degree to which SC migration was impacted by CTS. Thus, in both groups seeding was conducted on the higher side of a membrane with pores of 8mm, which were given free serum in a serum-free medium from the internal chambers at the bottom of the device. As shown in Figure 2, the cells that migrated from one side of the membrane to the other were counted, and it was observed that the frequency of migrated cells was higher in the 5% CTS group (*P<*0.05).

**Figure 3:**
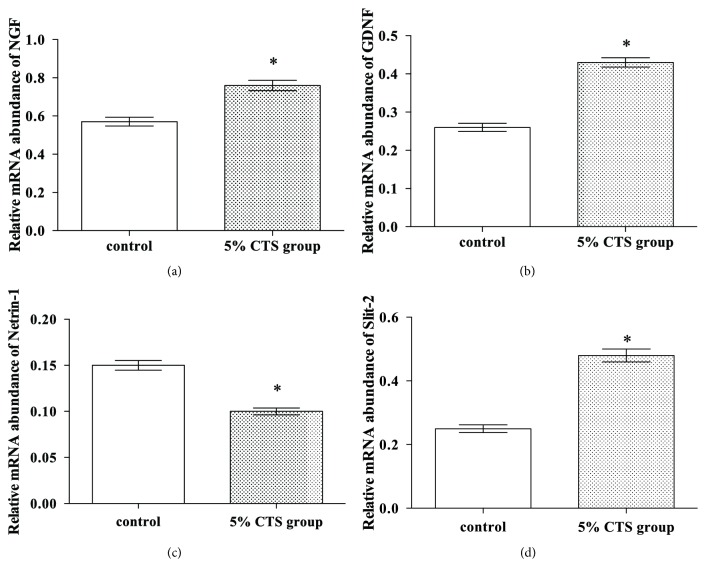
**Real-Time PCR **for (a) NGF, (b) GDNF, (c) Netrin-1, and (d) Slit-2 gene expression for the control and 5% CTS groups. *∗* indicates that 5% CTS group differs significantly from the control group (*P*<0.05).

The final analytical operation conducted was to examine the degree to which the neurotrophic function of SCs was affected in the 5% CTS group. In order to do so, Real-Time PCR for the quantitative evaluation of target gene expression (namely, those involved in neuroregeneration: NGF, GDNF, Netrin-1, and Slit-2) was utilised for both groups (Figure 3). The results indicated that the mRNA expressions for NGF, GDNF, and Slit-2 were significantly upregulated for the 5% CTS group—more so than for the counterpart control group (*P<*0.05). Additionally, Netrin-1 mRNA expression was considerably reduced for the 5% CTS group in comparison to the control group (*P<*0.05).

**Figure 4:**
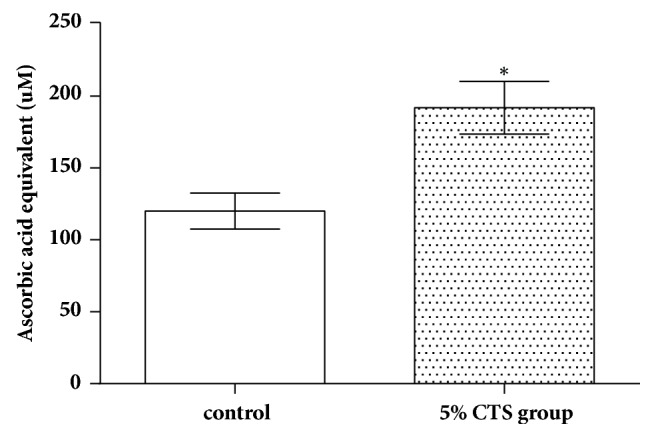
Total antioxidant capacity in Schwann cells. *∗* indicates that 5% CTS group differs significantly from the control group (*P<*0.05).

**Figure 5:**
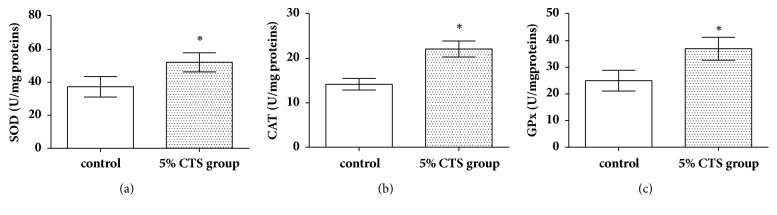
Activities of antioxidant enzymes in Schwann cells. *∗* indicates that 5% CTS group differs significantly from the control group (*P<*0.05).

The ABTS assay was to examine the total antioxidative capacity of SCs (Figure 4). The results indicated that the total antioxidative capacity of SCs were significantly upregulated for the 5% CTS group than that in the control SCs group (*P<*0.05). Meanwhile, the activities of antioxidant enzymes were determined (Figure 5). The results showed the SOD, CAT, and GPx were significantly upregulated for the 5% CTS group than that in the control SCs group (*P<*0.05).

## 4. Discussion

The significance of this research stems from the fact that it represents the initial systematic examination of the impact that (5%) CTS has on SCs in terms of migration and mRNA expression. The results indicated that moderate loading of CTS caused histological alteration, increase in SC migration, and upregulation in NGF, GDNF, and Slit-2 mRNA expression

Research has demonstrated that the proper operation of the PNS is predicated on the synaptic connections between neurons, which subsequently creates an intercellular functional network [17]. Axonal guidance is a critical type of movement engaged in by neuronal axons, and it occurs when they are exposed to specific stimuli [18]. In the context of the neuroregeneration of peripheral nerves, the critical stages are remyelination and axonal extension, and it is important to recognise that both are facilitated by SCs. Filamentous pseudopodal extension or recovery is initiated by the neural growth cone skeletal eggs during the preliminary phases of neuroregeneration. This arises as a consequence of the activation of attractant (Netrin family) or excitatory (Slit family) axon guidance factors—the most widely known cases of which are slit, netrin, ephrin, semaphoring, and Rgm—in combination with its receptor. In turn, this causes effective synaptic engagement with target cells [18]. Previous academic research has primarily focused on the Slit-2 and Netrin-1 genes in each of their respective classifications [19, 20]. Of significant relevance to the present study is the observation reported by Li et al. [21], who determined that a critical process in the preliminary phase of neural repair is SC migration; it is crucial not only in the context of axonal regeneration but also in neuroregeneration.

As previously noted, the body of literature addressing the impact that mechanical stimuli have on SC proliferation and expression is not extensive. Henninger et al. established a correlation between an increase in the propagation of SC and the expression of the Laminin gene and periodic tensile stress loaded on SCs [22], and Zhang et al. observed a relationship between CTS and the promotion of SC-induced BDNF secretion [23]. The latter finding is significant, because this phenomenon has been associated with the promotion of the axonal regeneration of SCs, thereby indicating that mechanical stress could have an impact on the establishment of the cytoskeleton. Despite the publication of findings such as these, no insights into the impact that CTS has on the expression of the nerve-guiding factors of SCs can be found in the existing literature.

The findings reported in this paper demonstrated that after a duration of 12 hours, 5% CTS corresponds to a polar configuration regarding the morphological features of SCs. It is noteworthy that the direction of this phenomenon is comparable to the direction of cyclic stretch stress. Moreover, assessment of microchemotaxis illustrated the capacity for SC migration after 5% CTS, and the findings showed that 5% CTS substantially heightened the frequency of migrated SCs. Both SC groups were evaluated regarding neural factors so as to better ascertain the functionality of SCs. One of the primary discoveries was that SCs supported the expression and secretion of NGF and GDNF as neurotrophic factors. It is important to further investigate these neurotrophic factors, as they carry out critical biological roles supporting the peripheral neuron subsistence and protection [24]. When compared to the control group, the gene expression of NGF and GDNF was significantly higher for the 5% CTS group; furthermore, the downregulation of Netrin-1 expression in SCs was substantial and the upregulation of Slit-2 expression was observed. Nevertheless, the impact of mechanical stimulation regarding Slit-2 secretion by SCs* in vitro *was considerably greater than Netrin-1. At a certain level, the directional growth pattern of axons in this context is dominated axon repulsion (in contrast to axon outgrowth), while at another level, the temporal and frequency variables associated with CTS (in combination with additional variables) may affect SC expression.

The findings illustrate that moderate CTS has the potential to lead to the effective stimulation of SCs, and cytoskeleton remodelling and alterations were observed to arise due to a related inducement in the upregulation of nerve-oriented factors expression. Histologically, the modifications were associated with axillary guidance growth. However, further studies are needed to show how the CTS affects the SCs of the animals in vivo. Overall, the present study contributes to the existing academic literature by detailing the influence that appropriate mechanical conditions can have regarding the expression of axon guidance-related factors in SCs, thereby accounting for a gap in the extant literature. In this way, this research represents the first confirmation for the expression of axon guidance factors of SCs under certain mechanical action by utilising the mechanical intervention of SCs.

## Data Availability

All the data are available at the corresponding author upon request.
